# An In Vitro Evaluation of the Effect of Nanoparticle Graphene Oxide on Compressive Strength of Conventional and Resin-Modified Glass Ionomer Cements (GICs)

**DOI:** 10.7759/cureus.113382

**Published:** 2026-07-25

**Authors:** Arushi Dhole, Neha Jaju, Sneha Rathaur, Pankaj Kumar Gupta, Shrenika Lall, Somraj Podder

**Affiliations:** 1 Department of Conservative Dentistry and Endodontics, Rungta College of Dental Sciences and Research, Bhilai, IND

**Keywords:** compressive strength, glass ionomer cements, nanographene, nanoparticles, resin-modified glass ionomer cement

## Abstract

Introduction: Conventional glass ionomer cements (CGICs) exhibit favorable properties, including chemical adhesion and fluoride release, but demonstrate limited mechanical strength, restricting their use in posterior stress-bearing restorations. Resin-modified glass ionomer cements (RMGICs) offer improved mechanical performance, yet remain inferior to resin composites. Nanotechnology, particularly graphene oxide incorporation, has emerged as a promising strategy to enhance the mechanical properties of dental cements. This in vitro study evaluated the effect of 2 wt% nanographene oxide (nGO) on the compressive strength of CGIC and RMGIC.

Material and methods: Eighty cylindrical specimens (6 mm × 4 mm) were fabricated and allocated to four groups (n = 20): CGIC control, CGIC + 2 wt% nGO, RMGIC control, and RMGIC + 2 wt% nGO. nGO powder was blended with cement powders at 2 wt% concentration and homogenized using an amalgamator. Specimens were stored at 37°C and 100% relative humidity for 24 hours. Compressive strength was measured using a universal testing machine at a crosshead speed of 1.0 mm/min. Data were analyzed using one-way ANOVA followed by Tukey's post hoc test (p < 0.05).

Results: ANOVA revealed significant differences among groups (p < 0.001, η² = 0.59). RMGIC + nGO demonstrated the highest mean compressive strength (104.10 ± 42.3 MPa), followed by RMGIC control (95.91 ± 36.69 MPa), CGIC + nGO (36.73 ± 10.27 MPa), and CGIC control (29.00 ± 6.11 MPa). RMGIC groups exhibited significantly higher compressive strength than CGIC groups (p < 0.001). However, within each material type, nGO incorporation did not yield statistically significant improvements (CGIC: p = 0.829; RMGIC: p = 0.802).

Conclusion: Incorporation of 2 wt% nGO did not significantly enhance compressive strength in either CGIC or RMGIC. RMGIC demonstrated substantially superior compressive strength compared to CGIC regardless of nGO modification. Further investigation with varied concentrations and long-term aging studies is warranted to optimize nGO reinforcement.

## Introduction

Replacement of the lost tooth structure is a fundamental component of comprehensive oral health care, especially in load-bearing areas where restorative materials must endure substantial occlusal forces while preserving the remaining tooth structure [1]. An ideal restorative material should therefore provide adequate mechanical strength, wear resistance, biocompatibility, adhesion to the tooth structure, dimensional stability, and the ability to prevent secondary caries. Fluoride release is an additional desirable property, as it contributes to remineralization, inhibits bacterial metabolism, and enhances the long-term success of restorations [2,3].

Conventional glass ionomer cements (CGICs) have been widely employed because of their chemical adhesion to enamel and dentin and sustained fluoride release [2,4]. These materials are used in atraumatic restorative treatment, cervical lesions, and pediatric restorations, as base/liner materials, and in patients with high caries risk, owing to their favorable physicochemical and anticariogenic properties [5]. However, CGICs exhibit intrinsic mechanical limitations including low compressive strength, brittleness, poor wear resistance, and susceptibility to fracture under occlusal loading, which restrict their use in high-stress-bearing posterior regions [6].

To address these shortcomings, resin-modified GICs (RMGICs) were developed by incorporating hydrophilic resin components into conventional formulations, producing a dual-cure system with both acid-base reaction and resin polymerization [6]. RMGICs demonstrate improved early strength, reduced moisture sensitivity, and better handling characteristics than CGICs. However, their flexural strength, fracture toughness, and wear resistance remain inferior to those of resin composites, limiting their durability in posterior load-bearing situations [7]. Other reinforcement approaches, such as metal-reinforced GICs and fiber reinforcements, have yielded only partial mechanical improvements, underscoring the need for alternative strategies [8].

Nanotechnology has emerged as a promising avenue for enhancing the mechanical and biological performance of GICs by incorporating nanoparticles into the cement matrix [9]. The addition of nanomaterials, such as nano-hydroxyapatite, nano-silica, and nano-titania, has been shown to improve the compressive and flexural strength, microhardness, and wear resistance by increasing the filler surface area and promoting stronger filler-matrix interactions, thereby improving stress distribution and reducing microcrack propagation under occlusal loading [10]. Importantly, these enhancements can often be achieved while maintaining the key clinical advantages of GICs, including chemical adhesion, fluoride release, biocompatibility, and acceptable handling characteristics.

Nanographene oxide (nGO), a derivative of graphene, has attracted considerable interest because of its exceptional mechanical properties, large specific surface area, and abundant oxygen-containing functional groups that favor strong interfacial bonding with polymeric and inorganic matrices [11]. nGO-based materials have demonstrated favorable biocompatibility and antibacterial effects, suggesting potential benefits in restorative and preventive dentistry [12]. Recent in vitro studies have indicated that the incorporation of graphene or graphene-based nanomaterials into GICs can significantly enhance shear bond strength, flexural strength, microhardness, and microleakage [13,14]. Although nGO has shown promising reinforcing properties, its influence on the compressive strength of CGIC and RMGIC has not been adequately investigated. Compressive strength was chosen as the primary outcome because it reflects the material's ability to withstand predominantly compressive occlusal loads during mastication, thereby ensuring clinically relevant load-bearing performance. Furthermore, as a widely reported parameter in nanoparticle-reinforced glass ionomer cement studies, its selection facilitates direct comparison of our findings with prior literature. Therefore, this in vitro study aimed to assess the effect of nGO incorporation on the compressive strength of CGIC and RMGIC.

## Materials and methods

Study design and setting

This in vitro experimental investigation was conducted in the Department of Conservative Dentistry and Endodontics, Rungta College of Dental Sciences and Research, Bhilai, Chhattisgarh, India, from May 2024 to December 2024, involving commercially available dental materials only. Therefore, approval from the institutional ethics committee was not required (Letter no. RCDSR/IEC/MDS/2024/D-90, dated 08-04-2024). A completely randomized four-group parallel design was employed to evaluate the effect of nGO reinforcement on the compressive strength of CGIC and RMGIC under standardized laboratory conditions.

Sample size estimation

A priori sample size estimation was performed using G*Power software (version 3.1.9.2; Heinrich Heine University, Düsseldorf, Germany). The calculation was based on a two-tailed test, assuming an effect size (Cohen's f = 0.40) derived from a previous study [15], a significance level (α) of 0.05, and a statistical power of 95%. This computation required 18 specimens per group (total n = 72) to detect clinically relevant differences in compressive strength. To compensate for the anticipated 10% attrition resulting from material loss during fabrication, visible surface defects, or accidental breakage during handling and storage, the total number of fabricated specimens was 80 (20 per group).

Inclusion and exclusion criteria

Specimens were included if they were cylindrical samples prepared from the specified commercial CGIC (GC Gold Label Universal Restorative, GC Corporation, Tokyo, Japan) and RMGIC (GC Gold Label Light Cured Universal Restorative, GC Corporation, Tokyo, Japan) materials in strict accordance with the manufacturers' instructions, incorporated exactly 2 wt% nGO in the designated experimental groups, and possessed standardized dimensions of 6 mm height and 4 mm diameter, free from any visible surface defects, voids, or irregularities.

Specimens were excluded if they exhibited air bubbles, surface defects, chipping, or dimensional inaccuracies following demolding, fracture, or sustained damage during fabrication, handling, storage prior to testing, or did not conform to the prescribed powder-liquid ratios or curing protocols established for the respective material types.

Preparation of graphene

nGO powder (Adnano Technologies Pvt. Ltd., Bengaluru, Karnataka, India) was used as the reinforcing nanofiller. According to the manufacturer's specifications, the nGO consisted of few-layer graphene oxide nanosheets, with a purity of ≥99.5%, an average lateral particle size of D50 < 10 μm, a thickness of 0.8-2 nm, and a specific surface area of 120 m^2^/g. These manufacturer-provided physicochemical characteristics were used as the reference for the material employed in this investigation. A digital analytical balance with an accuracy of 0.0001 g (Shimadzu Corporation, Kyoto, Japan; Model AUW220D) was utilized to obtain the precise quantities of nGO and glass ionomer powders at ambient room temperature (25 ± 1°C). For each nanographene-reinforced batch, 2 wt% nGO was blended with the corresponding cement powder; specifically, 5 g of CGIC powder was combined with 0.1 g of nGO to achieve a 2 wt% mixture, and the identical proportion (5 g base powder + 0.1 g nGO) was employed for the RMGIC powder formulation [16].

The nGO was initially mixed manually with the cement powder using an agate spatula to ensure preliminary dispersion, followed by further homogenization in an amalgamator (Dentsply Caulk, Milford, DE, USA; Model Vari-Mix III) for 20 s using an empty amalgam capsule, a process designed to promote a more uniform nanoparticle distribution within the powder phase through mechanical agitation.

Fabrication of specimens

Cylindrical specimens, measuring 6 mm in height and 4 mm in diameter, were fabricated using customized Teflon molds (6 mm height × 4 mm internal diameter; custom fabricated, Mumbai, Maharashtra, India) with precision-machined cavities. Prior to filling, the internal surfaces of the molds were lightly coated with petroleum jelly (Smith & Nephew Healthcare Pvt. Ltd., Mumbai, India) to facilitate the gentle removal of the set specimens without inducing damage. A Mylar strip (Dentsply Sirona, York, PA, USA; Model Dimension®) was positioned over the occlusal surface during the setting phase to obtain a smooth, flat finish and to eliminate surface irregularities. For Group I (CGIC control), one scoop of CGIC powder was mixed with one drop of the corresponding liquid on a glass mixing pad for approximately 25 s using an agate spatula at a mixing rate of one rotation per second until a homogeneous, glossy consistency was achieved. The mixed cement was subsequently placed into a Teflon mold and covered with a Mylar strip until completion of the setting reaction. For Group II (CGIC + 2 wt% nGO), the premixed CGIC + 2 wt% nGO powder was handled identically, with one scoop of the modified powder combined with one drop of the corresponding liquid for 25 s using the same mixing technique. The cement was then transferred into the molds, covered with a Mylar strip, and allowed to set completely. For Group III (RMGIC control), one scoop of RMGIC powder was mixed with two drops of liquid on the mixing pad for 25 s using a standardized spatulation technique. The material was placed into the mold, the top surface was covered with a Mylar strip, and light curing (3M ESPE, St. Paul, MN, USA; Model Elipar™ S10, wavelength 430-480 nm, intensity 1200 mW/cm²) was performed for a total of 60 s, delivered in three cycles of 20 s each, using a light curing unit positioned perpendicularly to the specimen surface at a distance of 2 mm. For Group IV (RMGIC + 2 wt% nGO), the RMGIC + 2 wt% nGO powder was mixed with two drops of liquid for 25 s and placed into the mold under a Mylar strip and light-cured for 60 s in three 20-second cycles as in Group III, followed by additional curing of the bottom surface after removal from the mold to ensure complete polymerization of the entire specimen volume. Following the setting process, all specimens were carefully removed from the molds using gentle pressure and visually inspected. Any specimens exhibiting defects, voids, or irregularities were discarded and remade under identical conditions to maintain sample integrity.

Storage conditions

All acceptable specimens meeting the inclusion criteria were stored in a controlled-environment Thermolab incubator (Thermolab Scientific, Mumbai, Maharashtra, India) maintained at 37°C (standard body temperature) and 100% relative humidity for 24 h to allow completion of the setting reaction and maturation processes prior to mechanical testing. The storage conditions were carefully maintained using a digital hygrometer and thermometer to ensure environmental stability throughout the maturation period and were placed in sealed containers containing distilled water to maintain saturated humidity levels and prevent desiccation of the glass ionomer materials.

Group assignment

Eighty specimens were fabricated and assigned to four predetermined experimental groups (n = 20 per group) according to the material formulation. Group I served as the control for the CGIC and comprised the CGIC without nGO reinforcement. Group II consisted of CGIC modified with 2 wt% nGO. Group III served as the RMGIC control and comprised the RMGIC without nGO. Group IV consisted of RMGIC modified with 2 wt% nGO. This grouping strategy enabled a comprehensive evaluation of both the effect of nGO incorporation within each material type and the inherent differences between CGIC and RMGIC formulations.

Testing of compressive strength

Compressive strength measurements were conducted using a universal testing machine (Instron Corporation, Norwood, MA, USA; Model 5965) equipped with a 5 kN load cell and Bluehill® Universal testing software version 3.0, following completion of the 24-hour incubation period. Each cylindrical specimen was positioned vertically between the upper and lower compression platens of the UTM, with the long axis of the specimen precisely aligned perpendicular to the platens, ensuring that the compressive load was applied coaxially along the longitudinal axis of the specimen. A compressive load was applied at a constant crosshead speed of 1.0 mm/min until catastrophic failure occurred, which was characterized by a visible fracture of the specimen and a sudden drop in the load-displacement curve. The maximum load at fracture (recorded in Newtons) was documented for each specimen using the integrated data-acquisition system of the instrument. The compressive strength values (expressed in megapascals, MPa) were subsequently calculated using the standard formula for cylindrical specimens:

\[ \text{Compressive Strength (MPa)} = \frac{\text{Maximum load (N)}}{\text{Cross-sectional area (A)}} \]

where the cross-sectional area (A) was derived from the known diameter (4 mm) of the specimen using the formula

\[ A = \pi \left(\frac{d}{2}\right)^2 \]

where: A = cross-sectional area (mm²); d = specimen diameter (mm); and π = mathematical constant (3.1416).

All specimens across the four experimental groups were tested under identical environmental conditions (ambient temperature 25 ± 1°C, relative humidity 50 ± 5%) to permit valid and reliable comparison of compressive strength values between groups.

Statistical analysis

All compressive strength data were systematically tabulated in Microsoft Excel (Version 2019; Microsoft Corporation, Redmond, WA, USA) and subsequently transferred to and analyzed using IBM SPSS Statistics for Windows, Version 24.0 (Released 2016; IBM Corp., Armonk, NY, USA). Continuous variables were summarized as the mean and standard deviation for each experimental group. One-way analysis of variance (ANOVA) was employed as the primary statistical test to compare the mean compressive strength among the four experimental groups (CGIC, CGIC + nGO, RMGIC, and RMGIC + nGO). When ANOVA revealed statistically significant differences between groups, Tukey's Honestly Significant Difference (HSD) post hoc test was applied for pairwise intergroup comparisons to identify which specific groups differed significantly from one another, while controlling for Type I error inflation. Statistical significance was set at p < 0.05, with a 95% confidence interval adopted for all inferential statistical analyses. The normality of the data distribution was assessed using the Shapiro-Wilk test to confirm the appropriateness of the parametric statistical methods.

## Results

One-way ANOVA (Table 1) revealed a highly statistically significant difference in mean compressive strength among the four experimental groups (p < 0.001), indicating that the material type and nGO incorporation collectively influenced the mechanical properties. The effect size (η² = 0.59) suggests that approximately 59% of the total variance in compressive strength could be attributed to group allocation, denoting a large magnitude of the treatment effect. RMGIC + nGO group (104.10 ± 42.3 MPa) demonstrated the highest mean compressive strength, followed by the RMGIC control (95.91 ± 36.69 MPa). In contrast, the CGIC groups exhibited substantially lower values, with the GIC + nGO group yielding 36.73 ± 10.27 MPa and the GIC control demonstrating the lowest mean strength of 29.00 ± 6.11 MPa.

**Table 1:** Intergroup comparison of compressive strength with one-way ANOVA test. F-statistics are calculated with a one-way ANOVA test; effect sizes are calculated as eta squared (η²). CGIC: conventional glass ionomer cement, RMGIC: resin-modified glass ionomer cement. *p < 0.05.

Group	N	Shapiro-Wilk test value (p-value)	95% CI for mean	Mean ± Std.	F-statistics	p-value	Effect size (η^2^)
GIC	20	0.98 (0.938)	26.14-31.86	29.00 ± 6.11	37.18	< .001*	0.59
GIC+ nanographene oxide	20	0.87 (0.364)	31.92-41.53	36.73 ± 10.27			
RMGIC	20	0.92 (0.116)	78.73-113.08	95.91 ± 36.69			
RMGIC + nanographene oxide	20	0.91 (0.055)	84.31-123.9	104.10 ± 42.3			

Tukey's post hoc pairwise comparisons demonstrated that both RMGIC groups (control and nGO-modified) exhibited significantly higher compressive strengths than both CGIC groups (Table 2), with all inter-category comparisons yielding p < 0.001. Specifically, the mean difference between CGIC and RMGIC was -66.91 MPa, while the difference between CGIC + nGO and RMGIC + nGO was -67.38 MPa. However, within each material category, the addition of 2 wt% nGO oxide did not result in a statistically significant enhancement in compressive strength; the comparison between CGIC and CGIC + nGO revealed a mean difference of -7.73 MPa (p = 0.829), and the comparison between RMGIC and RMGIC + nGO demonstrated a mean difference of -8.20 MPa (p = 0.802). These findings indicate that although the material type profoundly affects the compressive strength, the tested concentration of nGO did not produce a significant reinforcing effect within the constraints of this study.

**Table 2:** Post hoc Tukey analysis for pairwise group analysis. t-statistics are calculated with pairwise post hoc Tukey test. CGIC: conventional glass ionomer cement, RMGIC: resin-modified glass ionomer cement. *p < 0.05.

Pairwise group	Mean difference	Standard error	t-statistics	p-value	95% CI lower limit	95% CI upper limit
CGIC - CGIC+ nanographene oxide	-7.73	9.05	-0.85	0.829	-32.25	16.80
CGIC - RMGIC	-66.91	9.05	-7.39	<0.001*	-91.43	-42.38
CGIC - RMGIC + nanographene oxide	-75.10	9.05	-8.30	<0.001*	-99.63	-50.58
CGIC+ nanographene oxide - RMGIC	-59.18	9.05	-6.54	<0.001*	-83.71	-34.65
CGIC+ nanographene oxide - RMGIC + nanographene oxide	-67.38	9.05	-7.44	<0.001*	-91.90	-42.85
RMGIC - RMGIC + nanographene oxide	-8.20	9.05	-0.91	0.802	-32.72	16.33

## Discussion

The primary finding of this study was that the incorporation of 2 wt% nGO did not produce a statistically significant increase in compressive strength within either the CGIC or RMGIC groups. Both nGO-modified groups exhibited higher mean compressive strength values than their respective controls. The RMGIC + nGO group demonstrated the highest absolute compressive strength among all the groups, attributable primarily to the inherent superiority of the resin-modified formulation rather than the nGO reinforcement effect.

The substantial difference in the compressive strength between the CGIC and RMGIC groups warrants careful consideration. RMGIC formulations incorporate resin monomers alongside the traditional acid-base reaction, resulting in a more cross-linked polymer network that enhances the overall mechanical integrity of the set cement [6]. This dual-curing mechanism, involving both the conventional glass ionomer setting reaction and light-activated polymerization of resin components, produces a denser, more cohesive matrix capable of withstanding higher compressive loads [7]. The present findings corroborate this established understanding, with RMGIC groups demonstrating an approximately threefold higher compressive strength than their CGIC counterparts, irrespective of nGO modification.

The non-significant yet consistently positive trend in compressive strength following nGO incorporation merits further interpretation. The observed numerical improvements of 7.73 MPa in CGIC and 8.20 MPa in RMGIC suggest that nGO may exert a modest reinforcing effect that was insufficient to achieve statistical significance at the 2 wt% concentration under the current experimental conditions. This pattern is consistent with previous investigations reporting that nGO incorporation at lower concentrations often yields incremental improvements that may not reach statistical significance [11]. The proposed reinforcement mechanism of nGO in glass ionomer cement is related to its unique physicochemical properties. nGO possesses a two-dimensional lamellar structure with an exceptionally large specific surface area and abundant oxygen-containing functional groups, including hydroxyl and carboxyl moieties [12]. These functional groups can form hydrogen bonds and electrostatic interactions with the polyacrylic acid matrix and glass particles, potentially enhancing filler-matrix interfacial adhesion. When uniformly dispersed, nGO nanosheets may facilitate more effective stress transfer from the cement matrix to the reinforcing phase, thereby impeding crack propagation and improving overall mechanical performance [13]. However, the modest reinforcement observed in this study suggests that the 2 wt. % concentration may not have achieved optimal dispersion or interfacial bonding within either material system.

The relatively large standard deviations observed in the RMGIC groups suggest greater variability in compressive strength than in the conventional GIC groups. This variability may be attributed to the dual-setting mechanism of RMGIC, which combines the conventional acid-base reaction with light-activated resin polymerization. Minor differences in specimen fabrication, powder-liquid mixing, light-curing efficiency, or nanoparticle distribution may have contributed to greater inter-specimen variability. Consequently, although the mean compressive strength values were higher in the RMGIC groups, the increased variability may have reduced the statistical power to detect significant differences following nGO incorporation.

The differential response of CGIC and RMGIC to nGO incorporation warrants further consideration. The resin matrix in RMGIC may provide a more favorable environment for nanoparticle dispersion and interfacial bonding than the purely aqueous polyacrylic acid matrix of CGIC [17]. The polymerized resin network could potentially accommodate nGO nanosheets with reduced agglomeration, as the resin monomers may better wet the nanoparticle surface during the mixing and curing processes [11]. This hypothesis is supported by previous findings that nGO incorporation improves the flexural strength of RMGIC at 2 wt% concentration, suggesting more effective reinforcement within the resin-modified system [18]. Nevertheless, the present compressive strength data did not demonstrate a statistically significant advantage of nGO reinforcement in RMGIC over CGIC.

The concentration of 2 wt% nGO may not represent the optimal loading for maximizing the compressive strength. Previous studies have demonstrated that nGO concentrations below 1 wt% often yield minimal or statistically insignificant effects on mechanical properties, whereas concentrations of 3-5 wt% have been associated with significant improvements in the compressive strength of RMGIC [13]. However, higher nanoparticle concentrations are frequently accompanied by increased agglomeration, wherein the nanosheets cluster together rather than disperse uniformly throughout the matrix. Agglomeration creates stress concentration points that can serve as crack initiation sites, potentially negating the reinforcing benefits of nanoparticle incorporation [18]. The 2 wt% concentration employed in this study may therefore represent an intermediate loading that provides modest reinforcement without achieving the threshold required for statistical significance while simultaneously avoiding the detrimental effects associated with higher concentrations.

A comparison with previous nanoparticle reinforcement studies revealed both consistency and divergence. Research investigating fluorinated graphene incorporation in CGIC demonstrated significant improvements in compressive strength and microhardness at concentrations of 2 wt% [19]. Similarly, zirconium oxide and titanium dioxide nanoparticles have been shown to increase compressive strength when incorporated into GIC formulations [20]. However, the magnitude of reinforcement varies considerably depending on the nanoparticle type, concentration, dispersion methodology, and specific GIC formulation employed. The present findings align with studies reporting that nanographene oxide incorporation does not universally improve all mechanical properties; for instance, previous investigations have demonstrated significant improvements in flexural strength, microhardness, and bond strength without corresponding gains in compressive strength [13,14]. This discrepancy underscores the importance of evaluating multiple mechanical properties when assessing the potential for nanoparticle reinforcement.

The present study has several limitations. The in vitro design, while providing controlled experimental conditions, cannot fully replicate the complex oral environment characterized by cyclic loading, thermal fluctuations, pH variations, and enzymatic activity. The investigation employed only a single nGO concentration (2 wt%) and a single storage time point (24 h), precluding the assessment of concentration-dependent effects or long-term durability. The absence of aging protocols, thermocycling, or fatigue testing limits the extrapolation of these findings to clinical scenarios in which materials are subjected to prolonged cyclic loading. Although compressive strength is an important and clinically relevant mechanical property for restorative materials, it alone does not provide a comprehensive assessment of their overall mechanical performance. Furthermore, the relatively large standard deviations observed in the RMGIC groups suggest substantial variability in specimen fabrication and testing, which may have contributed to the failure to detect statistically significant differences.

Future research directions should encompass the systematic evaluation of multiple nGO concentrations to identify the optimal loading levels for compressive strength enhancement within each material system. Long-term aging studies incorporating thermocycling, water storage, and cyclic loading could provide valuable information regarding the durability of nGO reinforcements under simulated clinical conditions. Additionally, comprehensive characterization of the nGO dispersion within the cement matrix using scanning electron microscopy and energy-dispersive X-ray spectroscopy would elucidate the relationship between the nanoparticle distribution and mechanical performance. Investigations examining the effect of nGO on other clinically relevant properties, including fluoride release, bond strength to the tooth structure, wear resistance, and biocompatibility, would further inform the potential clinical utility of this modification strategy.

## Conclusions

In conclusion, within the limitations of this in vitro study, the incorporation of 2 wt% nGO did not result in a statistically significant improvement in the compressive strength of either CGIC or RMGIC; the consistent numerical increases observed suggest the potential for optimization through concentration refinement and improved dispersion techniques. The substantial superiority of RMGIC over CGIC in terms of compressive strength was confirmed, supporting the preferential use of resin-modified formulations in stress-bearing applications. nGO remains a potential reinforcing additive for glass ionomer cements, but further investigation is necessary to identify the optimal incorporation parameters and establish clinical relevance through comprehensive long-term evaluation.
